# Breeding progress, variation, and correlation of grain and quality traits in winter rye hybrid and population varieties and national on-farm progress in Germany over 26 years

**DOI:** 10.1007/s00122-017-2865-9

**Published:** 2017-03-13

**Authors:** Friedrich Laidig, Hans-Peter Piepho, Dirk Rentel, Thomas Drobek, Uwe Meyer, Alexandra Huesken

**Affiliations:** 1 Bundessortenamt, Osterfelddamm 80, 30627 Hanover, Germany; 2 Biostatistics Unit, Institute of Crop Science, University of Hohenheim, Fruwirthstrasse 23, 70599 Stuttgart, Germany; 3 Department of Safety and Quality of Cereals, Max Rubner-Institut, Federal Research Institute of Nutrition and Food (MRI), Schuetzenberg 12, 32756 Detmold, Germany

## Abstract

**Key message:**

Grain yield of hybrid varieties and population varieties in official German variety trials increased by 23.3 and 18.1%, respectively, over the last 26 years. On-farm gain in grain yield (18.9%) was comparable to that of population varieties in variety trials, yet at a level considerably lower than in variety trials. Rye quality is subject to large year-to-year fluctuation. Increase in grain yield and decline of protein concentration did not negatively influence quality traits.

**Abstract:**

Performance progress of grain and quality traits of 78 winter rye varieties tested in official German trials to assess the value for cultivation and use (VCU) were evaluated during 1989 and 2014. We dissected progress into a genetic and a non-genetic component for hybrid and population varieties by applying mixed models, including regression components to model trends. VCU trial results were compared with grain yield and quality data from a national harvest survey (on-farm data). Yield gain for hybrid varieties was 23.3% (18.9 dt ha^−1^) and for population varieties 18.1% (13.0 dt ha^−1^) relative to 1989. On-farm yield progress of 18.9% (8.7 dt ha^−1^) was considerably lagging behind VCU trials, and mean yield levels were substantially lower than in field trials. Most of the yield progress was generated by genetic improvement. For hybrid varieties, ear density was the determining yield component, whereas for population varieties, it was thousand grain mass. Results for VCU trials showed no statistically significant gains or losses in rye quality traits. For on-farm data, we found a positive but non-significant gain in falling number and amylogram viscosity and temperature. Variation of grain and quality traits was strongly influenced by environments, whereas genotypic variation was less than 19% of total variation. Grain yield was strongly negatively associated with protein concentration, yet was weakly to moderately positively associated with quality traits. In general, our results from VCU trials and on-farm data indicated that increasing grain yield and decreasing protein concentration did not negatively affect rye quality traits.

**Electronic supplementary material:**

The online version of this article (doi:10.1007/s00122-017-2865-9) contains supplementary material, which is available to authorized users.

## Introduction

Rye has been recognized to be relatively drought tolerant compared to other cereal crops (Schittenhelm et al. 2014). Therefore, rye is predominantly grown on infertile and sandy soils of the central and eastern parts of Europe, which are characterized by a low water holding capacity. Until 1960, winter rye was the main cereal crop in Germany, its acreage exceeding that of winter wheat. From 1960, the growing area of rye continuously dropped to 5–6% of total arable land in Germany today (DESTATIS). In 2014, 3.85 million tons of rye grain were harvested (StatJ 2015). 66% of national rye consumption was used for animal feeding and 15% for human nutrition, mainly for bread making (StatJ 2015).

In 1984, the first three hybrid varieties were released in Germany. Their higher yield potential, as compared to population varieties, allowed a fast adoption in practical farming. In 1994, already 44% of rye grain was harvested from hybrid and 56% from population varieties (Seibel and Weipert 1994). In 2014, about 81% of rye grain came from hybrids. Seven synthetic varieties were released between 1996 and 2014, of which the last was withdrawn in Dec. 2014. The share of population varieties, however, differs largely between regions; for example, in the federal state of Brandenburg with very sandy and low fertile soils, population varieties are in 32% of rye area, whereas in Niedersachsen, only 9% of the rye area is planted with population varieties (Muenzing et al. 2014).

Hansen et al. (2004) found a 10 to 20% higher harvest yield of hybrid varieties compared to population varieties. Miedaner and Huebner (2011) reported that hybrid breeding not only yielded more than 20 to 25% of higher grain yield as compared to population varieties, but also had further advantages: hybrid breeding allows to fix individual genotypes by continued selfing and to transfer monogenic traits into varieties. In addition, the selection of tested inbred lines with special quality characteristics is possible, which is of great importance for breeding towards better quality.

Grain yield can be dissected into the yield components kernels m^−2^, kernels ear^−1^, and thousand grain mass. The number of kernels per ear and ear density play an important role in winter rye yield progress, whereas thousand grain mass is of less importance for yield progress (Chmielewski and Koehn 2000; Peltonen-Sainio et al. 2007; Kottmann et al. 2016). Yield components are determined during different times of the growing season and, therefore, are subject to different conditions and stresses. Kernels ear^−1^ and ears m^−2^ are mainly determined by pre-anthesis growing conditions, but thousand grain mass is mainly governed by conditions during corn-filling (Peltonen-Sainio et al. 2007).

Contrary to wheat quality, where protein concentration and protein quality play a key role, for rye, alpha-amylase activity, starch, and pentosan concentration are the major quality determining compounds. Alpha amylase, as the main starch degrading enzyme, plays a key role in rye quality (Kucerova 2009). According to Weipert (1998b), starch and alpha-amylase activity are responsible for crumb elasticity of bread; pentosans influence water absorption and dough viscosity, which are functionally related to dough and bread volume. As compared with other cereal crops, rye has only a low secondary dormancy, which means that soon after morphological ripeness of the grain, germination processes may start if weather conditions are unfavorable (Bruemmer 2005). This process, called pre-harvest sprouting, induces increased alpha-amylase activity. Consequently, besides increasing yield, rye breeding has also been focused on reducing pre-harvest sprouting, because it negatively affects baking quality (e.g., Wehmann et al. 1991; Rattunde et al. 1994).

Harvested rye grain is considered to be of bread making quality according to the former EU intervention criterion (until 2003), if falling number >120 s, maximum amylogram viscosity (in the following referred to as amylogram viscosity) >200 AU, and amylogram temperature at maximum viscosity (in the following referred to as amylogram temperature) >63 °C (Muenzing et al. 2014). It is a well-known fact, reported in numerous studies, that rye quality is very different from year to year (e.g., Weipert 1998a, b; Chmielewski and Koehn 2000; Hansen et al. 2004; Bruemmer 2005; Kucerova 2009). The annual national total harvest share of rye with bread making quality according to the former EU intervention criterion varied between 25 and 100% during 1992–2014 with an average of 80% (Muenzing et al. 2014).

Only a few studies investigated and specified the relative influence of genotype and environment on grain and quality traits (Rattunde et al. 1994; Miedaner et al. 2012; Hansen et al. 2004; Kucerova 2009). Hansen et al. (2004), in a study with 19 different hybrid and population varieties grown at one location for up to 3 years, found that variation in grain yield and protein concentration was mainly due to genotypes, but that thousand grain mass and dietary fiber concentration was more strongly influenced by harvest year than by genotype. They further found that variation in starch properties, measured by falling number and amylogram peak temperature, were also more strongly influenced by harvest year.

Correlations between quality and grain traits are reported in many studies from different environments and genotypes (Weipert and Bolling 1979; Wehmann et al. 1991; Rattunde et al. 1994; Chmielewski and Koehn 2000; Hanson et al. 2004; Bruemmer 2005; Kucerova 2009; Miedaner et al. 2012). Obviously, results from different environments are not easily comparable. Nevertheless, these publications generally show that (1) moderate-to-strong positive relations exist between grain yield and ear density, between falling number and amylogram temperature, and between falling number and viscosity, but grain yield and protein correlate moderately to strongly negative, (2) no or weak correlations exist between thousand grain mass and yield, between protein concentration and falling number, and between amylogram viscosity and temperature.

The objective of our study is to (1) quantify progress of grain and quality traits in Germany, separately for hybrid and population varieties, and specify the part of progress caused genetically, (2) compare progress achieved in variety trials and on-farm, (3) evaluate the influence of genotypes and environment on variation of grain and quality traits, and (4) investigate phenotypic and genotypic correlations between traits.

## Materials and methods

### VCU trials and on-farm data

In official German variety trials, newly submitted candidate varieties are tested for their “value for cultivation and use (VCU)”. Each year about 20–30 new rye candidate varieties enter trials and are grown in three consecutive years at up to 25 locations per testing year with 2–3 replications before successful candidates are released. On average, only about three varieties were finally released each year, and trials were about equally distributed across the typical rye-growing regions in Germany during the studied period. Two different intensities of treatment were applied. Intensity two comprises the best local agronomic practice in fertilizer, fungicide, and other agrochemical treatment. For intensity 1, no fungicides and growth regulators were applied. Before 1990, only data from West German locations were available. We analysed only varieties registered for their VCU. Varieties which were withdrawn or rejected were eliminated from the data set. Seven synthetic winter rye varieties, tested between 1986 and 2006, have not been included. We eliminated this group, because its size is too small to estimate trends and other statistics separately for this group. Synthetics had no real impact on on-farm yield level and are of no further importance for farming today. At least three standards running in trials for several years were included. Well-established varieties were chosen as standards representing the actual state of breeding progress in agronomic and quality traits. A standard variety stays in trials about 7 years on the average, whereas a candidate varieties’ statutory testing period is 3 years. The oldest standard variety in this study was first tested in 1974. We used VCU data from intensity 2 which comprised 26 years (1989–2014) of trials grown at 45 different trial sites, and it contained 78 varieties, including 25 standards. 57 genotypes were hybrids and 21 population varieties. The number of observations per trait was between 3636 and 3794. The data set was very non-orthogonal covering only 3.22–3.38% of the possible variety-location-year combinations.

To assess indirect baking quality traits, bulked samples were taken at eight locations from intensity 2. Laboratory tests for VCU trials were carried out on behalf of the Bundessortenamt by the Department of Safety and Quality of Cereals, Federal Institute of Food and Nutrition, Detmold, Germany.

Studied grain and quality traits (Bundessortenamt 2015) are shown in Table 1. Grain yield, single ear density, and number of kernels ear^−1^ were measured at the same eight locations under the same intensity as the samples for the laboratory analysis. All other traits were assessed from laboratory grain samples. From 1992, thousand grain mass was assessed together with the other quality traits from laboratory samples.

**Table 1:** Investigated traits

Source	Trait	Abbreviation	Unit	Test type	Description
VCU	Grain yield at 86% dry matter	GRAIN_Y	dt ha^−1^	Field trial	Trial layout as split-plot, main plots in complete blocks (2 treatments), varieties in subunits. Average harvested plot size about 10 m^−2^
	Thousand grain mass at 86% dry matter	TGM	g (1000 kernels)^−1^	DIN EN ISO 520 (2010)	
	Single ear density	EAR_D	ears m^−2^	Field trial	Calculated from a row of one meter length of one single plot. All varieties at the same location are sown at equal number of kernels per square meter according to local conditions in the range from 200 to 500 kernels per square meter
	Number of kernels per ear	KERNELS_E	kernels ear^−1^	Field trial	Calculated from thousand grain mass and single ear density
	Falling number (Hagberg-Perten)	FALLING_N	s	DIN EN ISO 3093: (2009)	Describes the viscosity of a starch gel after fast gelatinisation and partial enzymatic starch degradation. A high falling number is an indicator for low alpha-amylase activity. It is influenced by pentosan content. Baking technology considers higher falling numbers as more favourable
	Crude grain protein concentration [% of dry matter]	PROTEIN_C	%	ICC 167 and ICC 159 with modified calibration for protein content	For bread rye, high values may reduce milling yield due to increased viscosity of kernels
	Maximum amylogram viscosity	AMYLO_V	AU	*ICC 126*/*1*	Most important method to assess properties of starch gelatinisation and consequently baking properties in winter rye. From the amylogram curve viscosity and temperature at its gelatinisation maximum is assessed. Low values for viscosity and temperature at maximum gelatinisation point are caused by high alpha-amylase activity and indicate an inelastic crumb and an all in all poor baking property, whereas temperature is of higher relevance than viscosity
	Amylogram temperature at maximum viscosity	AMYLO_T	°C		
On-farm	Grain yield at 86% dry matter	GRAIN_Y	dt ha^−1^		National average yield surveyed from on-farm winter rye harvests between 1989 and 2014 (StatJ 2015)
	Falling number (Hagberg-Perten)	FALLING_N	s	As for VCU	National averages from statutory annual harvest survey reports [“Besondere Ernte- und Qualitaetsermittlung (BEE)” 2014], kindly supplied by Department of Safety and Quality of Cereals, Federal Institute of Food and Nutrition, Detmold, Germany
	Crude grain protein concentration [% of dry matter]	PROTEIN_C	%	As for VCU	
	Amylogram viscosity	AMYLO_V	AU	As for VCU	
	Amylogram temperature	AMYLO_T	°C	As for VCU	

*AU* Amylogram unit

To avoid biased results, we checked VCU data thoroughly for consistent structure over time before carrying out analysis. Inconsistent data structures may have occurred due to changes in assessment of a characteristics’ scale of measurement or structure of trial series. The data were further checked for recording errors and outliers by calculating standardized residuals based on model (1), (2) and (3), as described in “Statistical analysis”. Observations with standardized residuals greater than ±5.0 were excluded from further analysis. Over all traits, 24 (0.08%) observations exceeded the threshold.

In contrast to winter wheat, winter rye varieties are not quality graded by a classification scheme before they are released. However, on the basis of falling number, protein concentration, and amylogram results, quality of newly released rye varieties is described according to a defined scoring scheme in the Descriptive Variety List (Beschreibende Sortenliste; Bundessortenamt 2015, page 73).

Unlike VCU data, the on-farm data set is based on national averages from statutory annual harvest survey reports [“Besondere Ernte- und Qualitaetsermittlung (BEE)” 2014] between 1989 and 2014, kindly supplied by the Department of Safety and Quality of Cereals, Federal Institute of Food and Nutrition, Detmold, Germany. The yield data include hybrid, population, and synthetic as well as mixtures between hybrid and population varieties. Quality traits falling number, amylogram viscosity, and temperature (Table 1) were assessed on representative harvest samples (761 samples 2014) as described by Muenzing et al. (2014).

### Statistical analysis

#### Model for genetic and non-genetic trend

We used the standard three-way model with factors genotype, location, and year given by (Laidig et al. 2008) and extended it to allow for different variety groups *l* (hybrid and population varieties):

1 $${{y}_{i(l)jk}}\,=\,{{\mu }_{l}}+{{G}_{i(l)}}+{{L}_{j}}+{{Y}_{k}}+{{\left( LY \right)}_{jk}}+{{\left( GL \right)}_{i(l)j}}+{{\left( GY \right)}_{i(l)k}}+{{\left( GLY \right)}_{i(l)jk}},$$

where *y*_*i*(*l*)*jk*_ is the mean yield of the *i*th genotype belonging to group *l* in the *j*th location and *k*th year, *µ*_*l*_ is the overall mean of the *l*th variety group, *G*_*i*(*l*)_ is the main effect of the *i*th genotype belonging to group *l, L*_*j*_ is the main effect of the *j*th location, *Y*_*k*_ is the main effect of the *k*th year, (*LY*)_*jk*_ is the *jk*th location × year interaction effect, (*GL*)_*i*(*l*)*j*_ is the *ij*th genotype × location interaction effect, (*GY*)_*i*(*l*)*k*_ is the *ik*th genotype × year interaction effect, and ${{\left( GLY \right)}_{i(l)jk}}$ is a residual comprising both genotype × location × year interaction and the sampling error arising from sampling the replications belonging to group *l*. Quality traits assessed on bulked laboratory samples are additionally subject to errors arising from laboratory processing. This model assumes that locations are crossed with years, i.e., at least some locations are used across several years. All effects except *µ, G*_*i*(*l*)_ and *Y*_*k*_ are assumed to be random and independent with constant variance for each effect. Genetic and non-genetic time trends were studied by modelling *G*_*i*(*l*)_ and *Y*_*k*_ with regression terms for time trends as follows (Laidig et al. 2014; Piepho et al. 2014a):

2 $${{G}_{i(l)}}={{\beta }_{l}}{{r}_{i}}_{(l)}+{{H}_{i(l)}},$$

where ${{\beta }_{l}}$ is a fixed regression coefficient for genetic trend of group *l, r*_*i*(*l*)_ is the first year of testing for the *i*th variety in group *(l)*, and *H*_*i*(*l*)_ models a random normal deviation of *G*_*i*(*l*)_ from the genetic trend line of group *l*, and

3 $${{Y}_{k}}=\gamma {{t}_{k}}+{{Z}_{k}},$$

where $\gamma$ is a fixed regression coefficient for the non-genetic trend which is assumed to be identical for both groups, *t*_*k*_ is the continuous covariate for the calendar year and *Z*_*k*_ is a random normal residual. Genetic and non-genetic trends are quantified by the regression coefficients *β*_*l*_ and *γ*, respectively, indicating the yield increase per year measured in the same units as *y*_*i*(*l*)*jk*_. It is assumed that variances of random effects in models (1) and (3) are homogeneous in different groups. Non-genetic trends are influenced by other than genetic effects, such as climate change and crop management.

#### Model for overall trend

Overall trend was modelled considering the genotype as nested within years (Laidig et al. 2014). Thus, compared with model (1), for this analysis, we dropped effects involving genotypes that are not nested within years, i.e., the effects *G*_*i*(*l*)_ and (*GL*)_*i*(*l*)*j*_. Consequently, we need to consider groupwise year effects. The reduced model is then given by

4 $${{y}_{i(l)jk}}={{\mu }_{l}}+{{L}_{j}}+{{Y}_{k(l)}}+{{\left( LY \right)}_{jk}}+{{\left( GY \right)}_{i(l)k}}+{{\left( GLY \right)}_{i(l)jk}}.$$

Similarly as in Eq. (3), the year main effect can be modelled as

5 $${{Y}_{k(l)}}={{\varphi }_{l}}{{t}_{k}}+{{U}_{k(l)}},$$

where ${{\varphi }_{l}}$ is a fixed regression coefficient for overall trend of group *l, t*_*k*_ is the continuous covariate for the calendar year, and *U*_*k*(*l*)_ is a random residual following a normal distribution with zero mean and variance $\sigma _{U}^{2}$. We take the year main effects as fixed to obtain adjusted means for years, representing the groupwise overall trend.

#### Model for national on-farm trend

To estimate on-farm trends from national harvest survey, we used the simple linear regression model:

6 $${{y}_{k}}=\mu +\omega {{t}_{k}}+{{e}_{k}}$$

where $\omega$ is a fixed regression coefficient for on-farm trend, *t*_*k*_ represents the calendar year, and *y*_*k*_ represents the average national on-farm result in year *k*.

#### Gain of performance from 1989 to 2014

To quantify the difference in performance level of individual traits at the beginning and at the end of studied period, we calculated the differences between the overall linear regression estimate of 1989 and 2014 and expressed the difference relative to overall regression at calendar year 1989 for hybrid and population varieties separately.

#### Genetic correlation

We estimated genetic correlation coefficients between traits by a univariate approach (Piepho et al. 2014b) as follows:

1. Calculate variance components according to the linear trend model (1), (2), (3) of trait (*p*) and (*q*) and for the difference between both traits.
2. Compute covariances between the genotypic effects ${{H}_{i}}_{(l)}$ (Eq. 2) from variance components obtained from univariate models by using the equation:

7 $$\begin{aligned} \text{var}\left(
{H}^{(p)}_{i(l)}-{H}^{(q)}_{i(l)}\right) &
=\text{var}\left({H}^{(p)}_{i(l)}\right)+\text{var}\left(
{H}^{(q)_{i(l)}}\right)\\ & -2\text{cov}\left(
{H}^{(p)}_{i(l)},{H}^{(q)}_{i(l)}\right) \\
&\Leftrightarrow \text{cov}\left(
{H}^{(p)}_{i(l)},{H}^{(q)}_{i(l)}
\right)\\&=\frac{\text{var}\left(
{{H}^{(p)}}{{_{i}}_{(l)}})+\text{var}({{H}^{(q)}}{{_{i}}_{(l)}}
\right)-\text{var}\left(
{{H}^{(p)}}{{_{i}}_{(l)}}-{{H}^{(q)}}{{_{i}}_{(l)}} \right)}{2}.
\end{aligned}$$

3. Use variances and covariance from (2) to estimate the correlation coefficient *ρ*.

#### Phenotypic correlation

To evaluate phenotypic correlation between traits, we considered effects for genotype *G*_*i*(*l*)_ and year *Y*_*k*_ to be fixed in model (1) and then calculated least square means for genotypes. We assessed correlation between traits by the Pearson correlation coefficient of least square means for genotypes. To avoid biased correlations due to different group means, we subtracted group means from genotype means and calculated correlation coefficients from pooled residuals.

#### Graphical displays

We define a fixed categorical effect ${{C}_{p}}_{(l)}$ for time class $p=1,\ldots ,P$, where $P$ is the number of levels of time variable ${{r}_{i}}$, where each class is represented by at least one genotype in group *l*. Then, the genetic effect can be modelled as

8 $${{G}_{i}}_{(l)}={{C}_{p(l)}}+H_{i(l)}^{\prime },$$

where $H_{i(l)}^{\prime }$ is the random deviation from categorical effect ${{C}_{p}}_{(l)}$. We compute adjusted means for ${{C}_{p}}_{(l)}$ and plot them against first year of testing (${{r}_{i}}_{(l)}$).

The plots used are based on the proposed models, as described in Table 2.

**Table 2:** Graphical displays of VCU and of on-farm results

Description	Ordinate	Abscissa	Equations used	Figures
Visible genetic group trends	Adjusted genotype class means *C*_*p*(*l*)_	Year of first testing *r*_*i*_	Equation (8) inserted in baseline model (1) keeping *C*_*p*(*l*)_ and *Y*_*k*_ fixed	Figure 1, column 1 and 2
Visible agronomic trends	Adjusted year means for *Y*_*k*_	Calendar (harvest) year *t*_*k*_	Equation (8) inserted in baseline model (1) keeping *C*_*p*(*l*)_ and *Y*_*k*_ fixed	Figure 1, column 1 and 2
Visible overall trends	Adjusted year means for *Y*_*k*(*l*)_	Calendar (harvest) year *t*_*k*_	Model (4) keeping *Y*_*k*(*l*)_ fixed	Figure 1, column 3
Visible on-farm trends	Average national year means *y*_*k*_	Calendar (harvest) year *t*_*k*_	Model (6)	Figure 1, column 3
Genotype by year plots	Adjusted genotype means *G*_*i*(*l*)_	Year of first testing *r*_*i*_	Model (1) keeping effects for genotypes *G*_*i*(*l*)_ and years *Y*_*k*_ fixed	Figure 2
Correlation plots	Adjusted genotype means *G*_*i*(*l*)_	Adjusted genotype means *G*_*i*(*l*)_	Model (1) keeping effects for genotypes *G*_*i*(*l*)_ and years *Y*_*k*_ fixed	Figure 4, Electronic Appendix Fig. S1

**Fig. 1:**
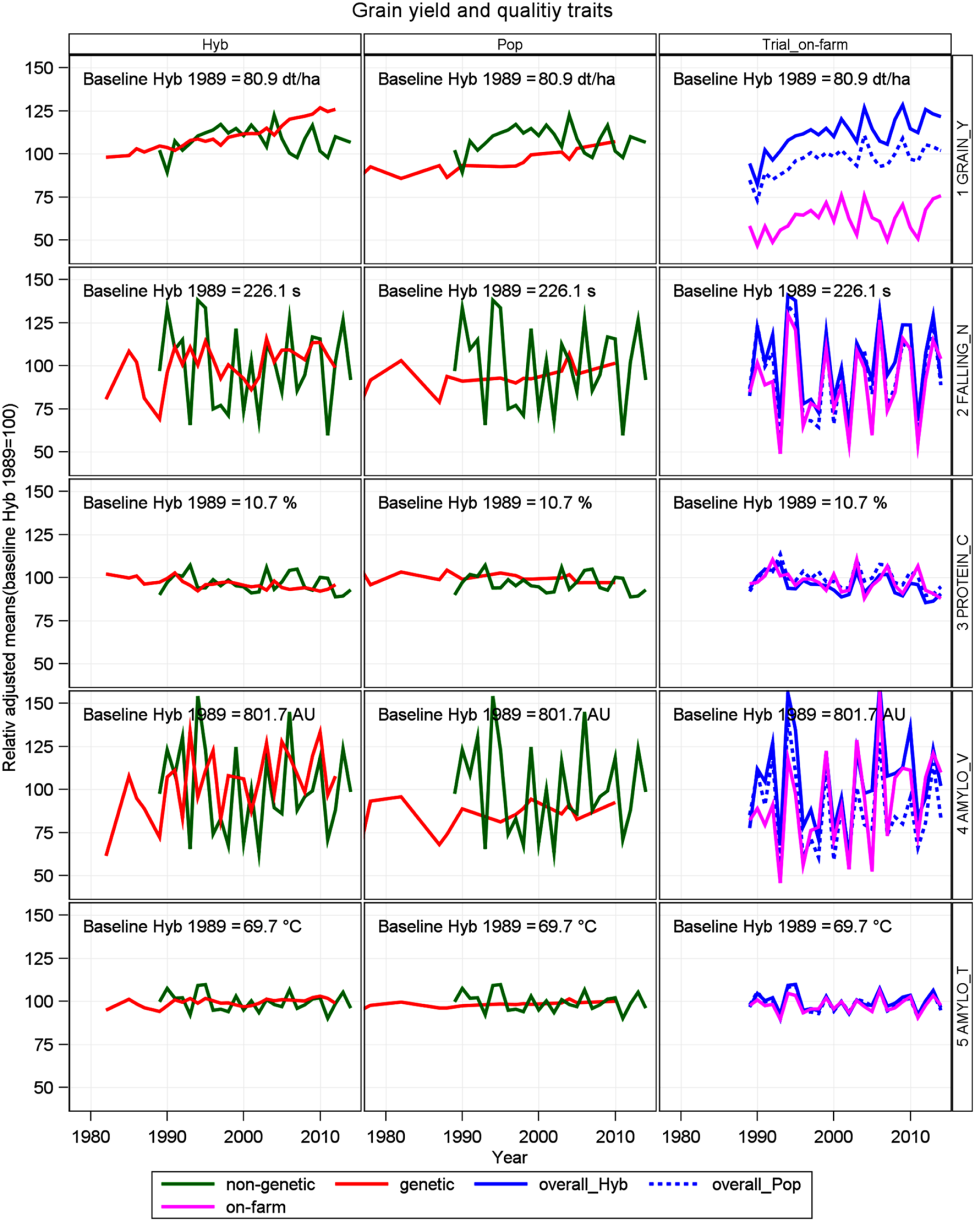
Relative adjusted means for grain yield and quality traits as percent of 1989 overall baseline for hybrid varieties. *GRAIN_Y* grain yield, *FALLING_N* falling number, *PROTEIN_C* crude protein concentration, *AMYLO_V* amylogram viscosity, *AMYLO_T* amylogram temperature, *AU* amylogram unit, *Hyb* hybrid varieties, *Pop* population varieties. Genetic: variety group means [effect ${{C}_{p}}_{(l)}$ in Eq. (8)]. Non-genetic: year means [Eq. (1), using Eq. (8) to model *G*_*i*(*l*)_ analog]. Overall: overall year means [effect Y_*k*(*l*)_ in Eq. (4)]. On-farm: annual national harvest survey [*y*_*k*_ in Eq. (6)]

**Fig. 2:**
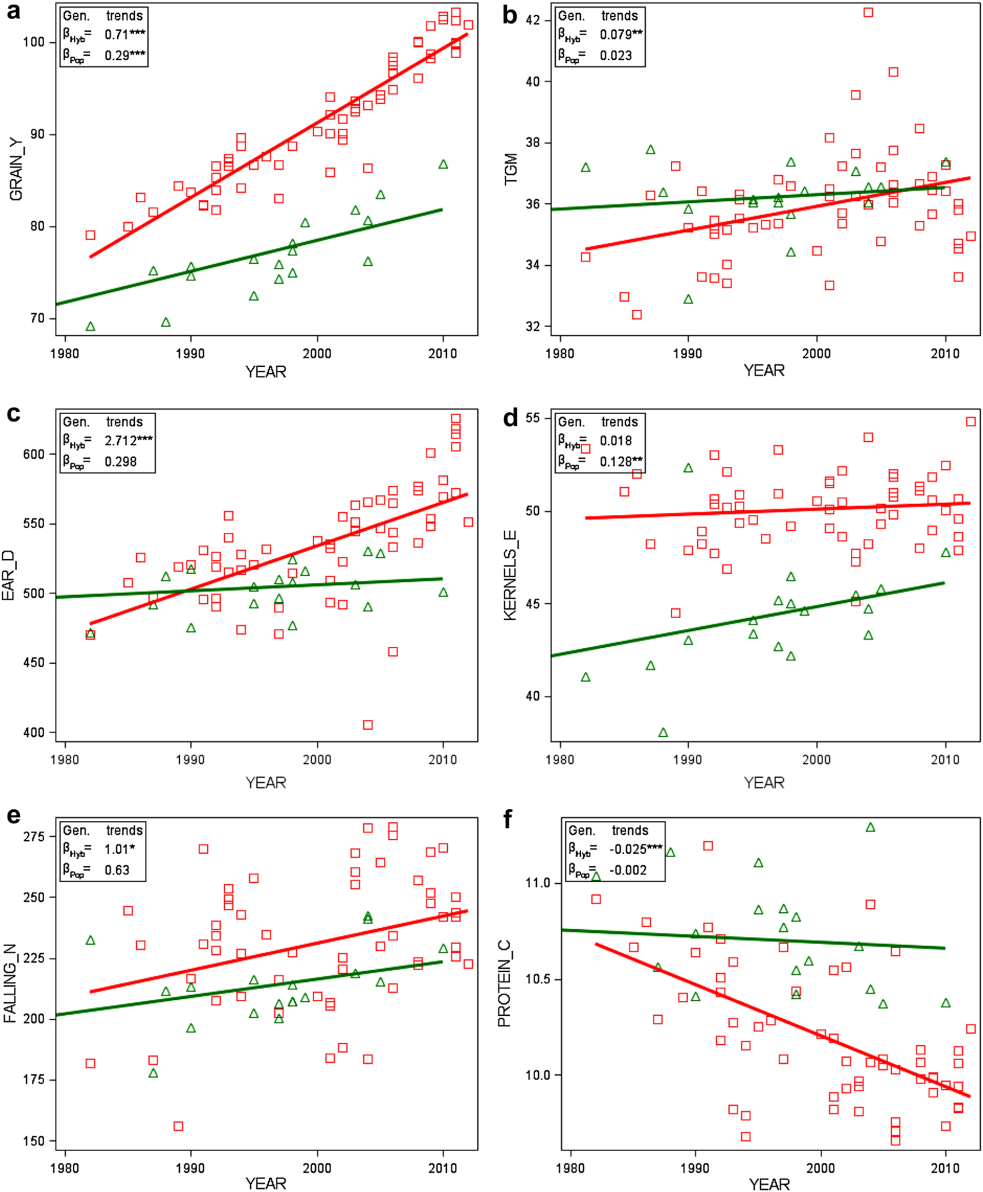

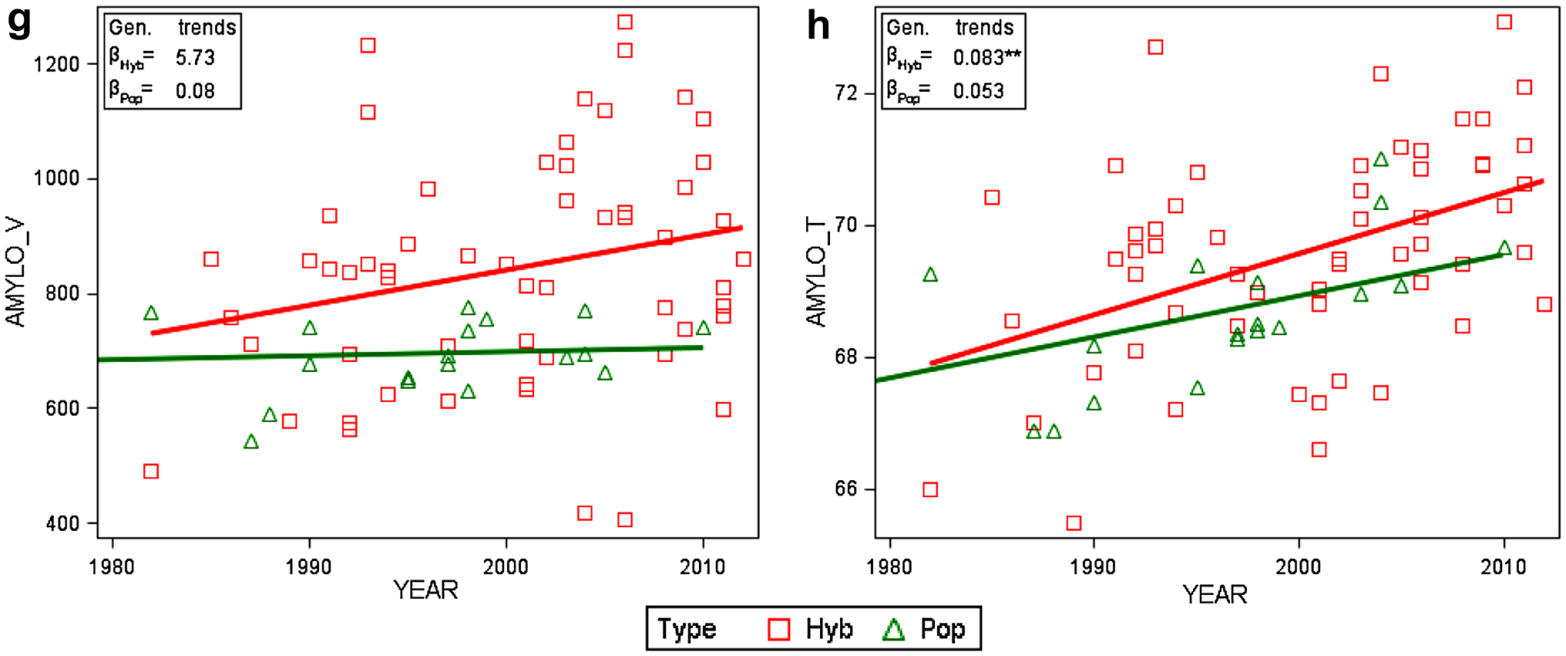
Adjusted variety means [effect ${{G}_{i(l)}}$ in Eq. (1)] plotted against first year in trial with group regression lines (number of varieties *n*_Hyb_ = 57 and *n*_Pop_ = 21). *YEAR* first year in trial, *GRAIN_Y* grain yield, *TGM* thousand grain mass, *EAR_D* single ear density, *KERNELS_E* number of kernels per ear, *FALLING_N* falling number, *PROTEIN_C* crude protein concentration, *AMYLO_V* amylogram viscosity, *AMYLO_T* amylogram temperature, *Hyb* hybrid varieties, *Pop* population varieties. β_Hyb_, β_Pop_: genetic trends [Eq. (1) using Eq. (2)]. *Significant at 5% level; **Significant at 1% level; ***Significant at 0.1% level

## Results

### Performance progress

#### VCU trials

In Table 3, we compare the gain or loss achieved in VCU trials separately for hybrid and population varieties, and on-farm expressed as the difference between overall trend 2014 and 1989. Furthermore, genetic, non-genetic, overall, and on-farm trends are shown in Table 3 and graphically displayed in Fig. 1 for grain yield and quality traits. In Fig. 2, adjusted variety means are plotted against the varieties’ first year in trial, and regression lines are shown for hybrid and population varieties. We assume a common non-genetic trend for both variety types, because both types are grown in the same trial receiving identical treatment.

**Table 3:** Estimates of regression coefficients of grain and quality traits

Traits		Source	Type	Overall regression estimates					Test of parallel linear genetic trends^a^		Estimates of linear trends											
											Genetic				Non-genetic				Overall			
Short name	Unit			1989	2014	Difference	Sign.	%Difference	*F* value	*P* value	Absolute	Sign.	SE	%	Absolute	Sign.	SE	%	Absolute	Sign.	SE	%
GRAIN_Y	dt ha^−1^	Trial	Hyb	80.9	99.8	18.9	***	23.3	39.13	<0.001	0.710	***	0.057	0.88	0.072		0.165	0.09	0.755	***	0.156	0.93
			Pop	71.7	84.7	13.0	**	18.1			0.295	***	0.056	0.40				0.10	0.520	**	0.162	0.73
		On-farm		45.9	54.6	8.7	*	18.9											0.348	*	0.170	0.76
TGM	g	Trial	Hyb	32.8	38.6	5.8	***	17.7	1.68	0.196	0.079	**	0.030	0.24	0.154	*	0.070	0.47	0.229	***	0.068	0.70
			Pop	33.5	39.0	5.5	**	16.5			0.023		0.036	0.07				0.46	0.221	**	0.071	0.66
EAR_D	m^−2^	Trial	Hyb	493.4	564.2	70.8	*	14.4	8.14	0.004	2.712	***	0.654	0.55	0.337		1.353	0.07	2.833	*	1.271	0.57
			Pop	505.1	515.7	10.6		2.1			0.298		0.698	0.06				0.07	0.424		1.326	0.08
KERNELS_E	ear^−1^	Trial	Hyb	53.5	44.7	−8.8		−16.4	3.67	0.055	0.018		0.052	−0.04	−0.150		0.114	−0.61	−0.131		0.108	−0.66
			Pop	45.3	41.6	−3.7		−8.2			0.128	**	0.048	0.30				−0.72	0.025		0.113	−0.33
FALLING_N	s	Trial	Hyb	226.1	234.8	8.7		3.8	0.32	0.570	1.009	*	0.463	0.45	−0.455		1.452	−0.20	0.347		1.470	0.15
			Pop	212.1	216.0	3.9		1.8			0.627		0.549	0.30				−0.21	0.156		1.495	0.07
		On-farm		195.6	213.9	18.3		9.4											0.732		1.383	0.37
PROTEIN_C	%	Trial	Hyb	10.7	9.7	−1.0	**	−9.7	7.25	0.007	−0.0247	***	0.0064	−0.230	−0.0166		0.0164	−0.155	−0.0415	**	0.0160	−0.386
			Pop	10.9	10.4	−0.5		−4.5			−0.0019		0.0070	−0.018				−0.152	−0.0197		0.0165	−0.180
		On-farm		10.8	10.1	−0.7		−6.4											−0.028		0.017	−0.26
AMYLO_V	AU	Trial	Hyb	801.7	881.2	79.4		9.9	1.33	0.248	5.735		3.399	0.72	−2.146		5.876	−0.27	3.178		5.634	0.40
			Pop	746.9	653.7	−93.2		−12.5			0.080		4.010	0.01				−0.29	−3.729		5.984	−0.50
		On-farm		616.4	860.7	244.3		39.6											9.773		5.386	1.59
AMYLO_T	°C	Trial	Hyb	69.7	69.1	−0.6		−0.8	0.60	0.438	0.083	**	0.029	0.12	−0.088		0.098	−0.13	−0.023		0.097	−0.03
			Pop	69.3	68.3	−0.9		−1.4			0.053		0.032	0.08				−0.13	−0.038		0.098	−0.06
		On-farm		68.0	68.4	0.4		0.5											0.015		0.079	0.02

Overall per cent trends (%) and per cent differences (%) relative to 1989 overall regression estimates

*GRAIN_Y* grain yield, *TGM* thousand grain mass, *EAR_D* single ear density, *KERNELS_E* number of kernels per ear, *FALLING_N* falling number, *PROTEIN_C* crude protein concentration, *AMYLO_V* amylogram viscosity, *AMYLO_T* amylogram temperature, *AU* amylogram unit, *Hyb* hybrid varieties, *Pop* population varieties, *VCU* on-farm: annual national harvest survey, *SE* standard error

*Significant at 5% level

**Significant at 1% level

***Significant at 0.1% level

^a^Test of parallel linear genetic type trends, *H*_0_:${{\beta }_{l}}={{\beta }_{l'}}$

*Grain traits* The test of parallel linear genetic trends indicated significantly different slopes between hybrid and population varieties for grain yield and single ear density, whereas for thousand grain mass and kernels ear^−1^, genetic trends were not significantly different (Table 3). Grain yield for hybrid varieties increased by 23.3% (18.9 dt ha^−1^) and for population varieties by 18.1% (13.0 dt ha^−1^) relative to 1989. In 1989, yield level for population varieties was 71.7 dt ha^−1^ and for hybrid varieties 80.9 dt ha^−1^. However, the yield gap between both types widened considerably to 15.1 dt ha^−1^ in 2014 (Table 3). Figure 2a demonstrates the enormous progress in grain yield achieved by hybrid varieties. Ear density of hybrids increased considerably due to genetic improvement with 14.4% (70.8 ears m^−2^) as compared to population varieties with only 2.1% (10.6 ears m^−2^, not significant) relative to 1989 (Table 3). Thousand grain mass increased by a slightly higher rate in hybrids than in population varieties. Overall regression estimates in 2014 indicated that thousand grain mass of hybrids (38.6 g) reached about the same level as of population varieties (39.0 g). Kernels ear^−1^ declined twice as much [−16.4% (−8.8 kernels ear^−1^)] for hybrids as compared to population varieties [−8.2% (−3.7 kernels ear^−1^)] relative to 1989 (Table 3).

*Quality traits* For falling number, amylogram viscosity, and temperature, we found no significant diverging genetic trends, except for protein concentration (Table 3). All non-genetic trends were not significantly different from zero (Table 3). As expected, protein concentration was decreasing in both types due to the well-known negative relationship with grain yield in cereals (Fig. 2 f). Only for hybrid varieties, we found a significant loss for protein concentration of −9.7% (−1.0% absolute change). Gains and losses for falling number, amylogram viscosity, and temperature were not significantly different from zero due to the large year-to-year fluctuations, as indicated by Fig. 1.

#### On-farm performance

When comparing on-farm with VCU results, we should keep in mind that on-farm data contain all variety types, including synthetics, which we dropped from the VCU data set in this study. In addition, we should consider that newer hybrid varieties may be introduced on-farm with a delay as compared to their testing period in VCU trials. Levels of grain yield for hybrid and population varieties show progress on a much higher level than on-farm (Table 3; Fig. 1). Though on-farm yield gain of 18.9% (8.7 dt ha^−1^) is of about the same magnitude as that of population varieties relative to 1989, but expressed in absolute figures, yield gain in VCU trials was considerably higher, for population (13.0 dt ha^−1^) and for hybrid rye (18.9 dt ha^−1^). On-farm decline of protein concentration of −6.4% (−0.7% absolute change) was intermediate to hybrid and population rye relative to 1989 and not significant. Gain in on-farm falling number of 9.4% (18.3 s) relative to 1989 was higher than in VCU trials, but not significant. Amylogram viscosity trends are not significant even for gains as large as 39.6% (244.3 AU) relative to 1989 caused by a very large year-to-year fluctuation. For amylogram temperature, a slight positive gain (0.5% relative to 1989) was found, yet not significant. In general, Table 3 indicates that on-farm quality trends have improved more than in VCU trials.

### Genotype, environment, and genotype by environment interaction

As shown in Table 3, hybrid- and population-type varieties are responding differently with respect to yield level and trends. To obtain unbiased estimates for variance components, we, therefore, modelled data by allowing for heterogeneity between groups in genetic trends ${{\beta }_{l}}$, overall trends ${{\varphi }_{l}}$, and overall-means ${{\mu }_{l}}$ (Eqs. 1, 2, 4). Variance components for the genotypic effect *H*_*i*(*l*)_ and the year effect *Z*_*k*_ are then unbiased deviations from linear trends, pooled within groups.

**Fig. 3:**
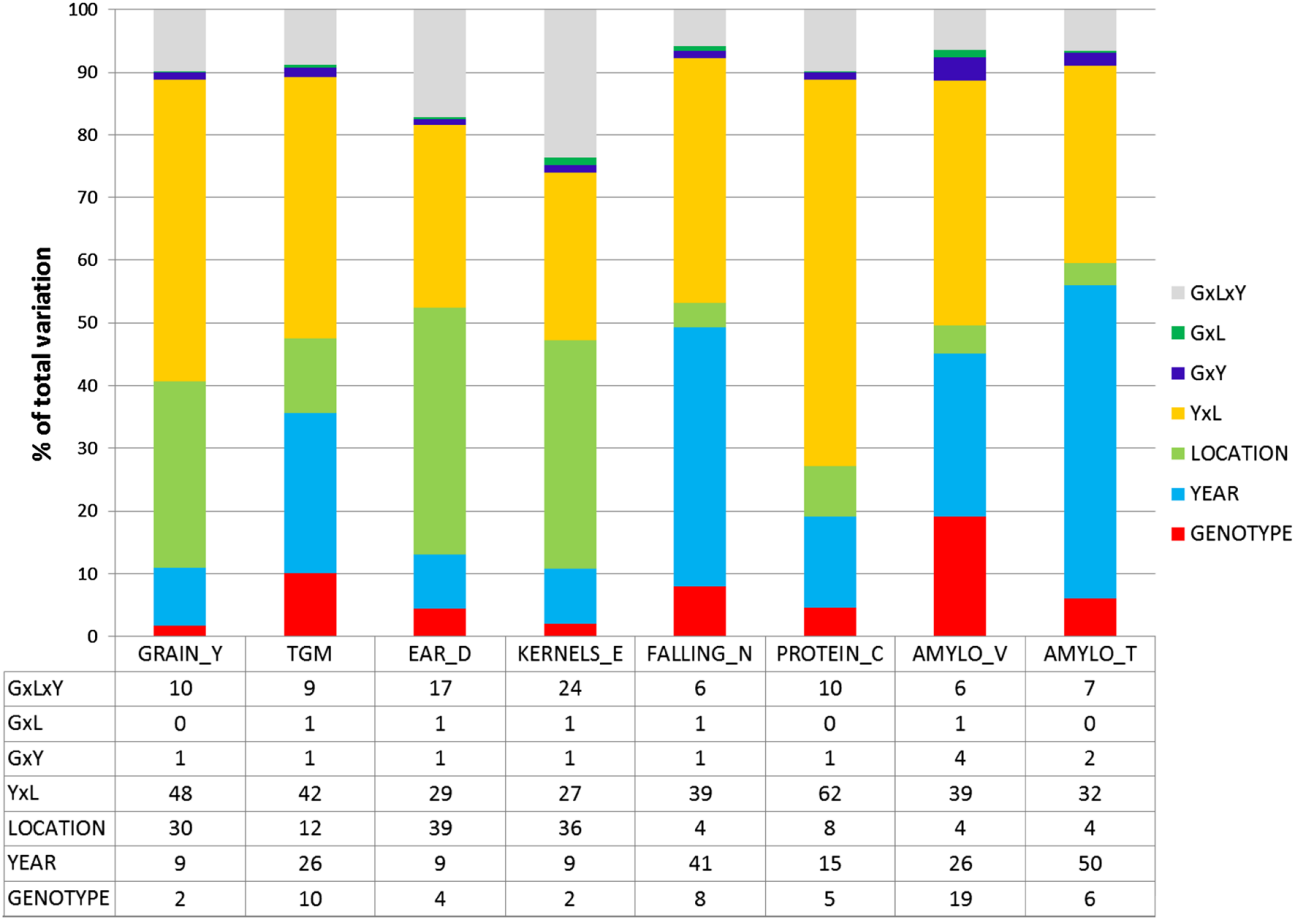
Sources of variation of grain and quality traits within hybrid and population varieties after elimination of genetic and non-genetic trends as percentage of total variability [Eq. (1), using Eqs. (2) and (3)]. *GRAIN_Y* grain yield, *TGM* thousand grain mass, *EAR_D* single ear density, *KERNELS_E* number of kernels per ear, *FALLING_N* falling number, *PROTEIN_C* crude protein content, *AMYLO_V* amylogram viscosity, *AMYLO_T* amylogram temperature

It is useful and illustrative to express variance components as percentage of their total sum (Fig. 3). Due to the large data set, variance components for genotype, year × location, genotype × year, and genotype × year × location were significantly greater than zero at p < 0.01 in this study. Most of the other variance components were also significant at *p* < 0.01 (variance components and their significance level are shown in Electronic Appendix Table S1).

Winter rye grain and quality traits are influenced to a great extent by environmental factors (year, location, and year × location interaction). Environmental variation was between 72 (kernels ear^−1^) and 87% (grain yield) as per cent of total variation. Year-to-year fluctuation dominates variation from location to location for amylogram temperature (50 vs 4%), falling number (41 vs 4%), amylogram viscosity (26 vs 4%), thousand grain mass (26 vs 12%), and protein concentration (15 vs 8%) (Fig. 3). In contrast, variance components of location effects for ear density (39%), kernels ear^−1^ (36%), and grain yield (30%) are greater than their corresponding year components. Genotypes have a minor influence on total variation in the range of 19% for amylogram viscosity and 2% for grain yield and kernels ear^−1^. Variation due to genotype × year and genotype × location interaction effects is of diminishing size, except for amylogram viscosity, where interaction with years reached 4% (Fig. 3).

### Phenotypic and genetic correlation

**Fig. 4:**
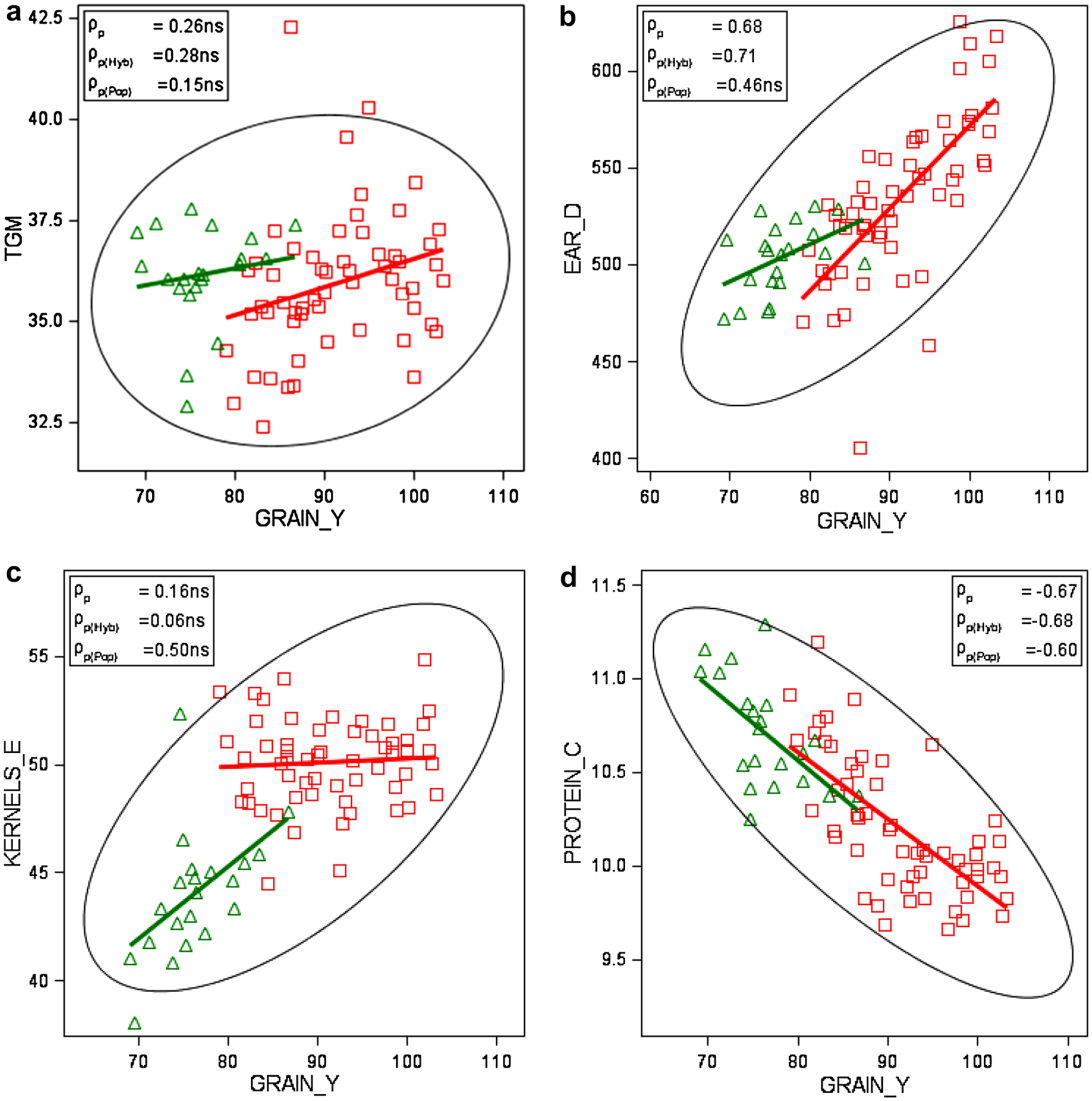

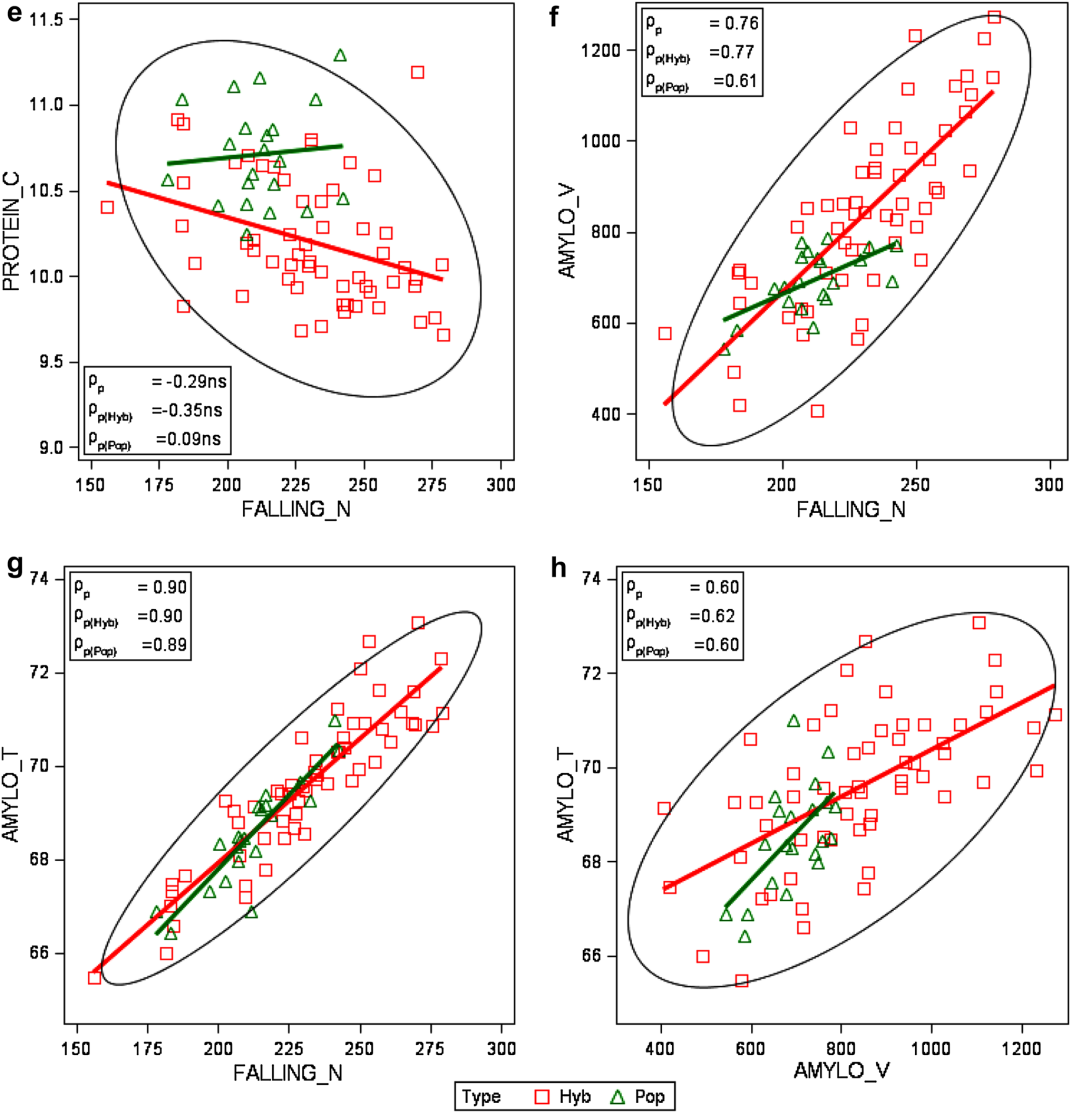
Phenotypic correlation of adjusted variety means [effect ${{G}_{i(l)}}$ in Eq. (1)] grouped by type of variety, with group regression lines (number of varieties *n*_Hyb_ = 57 and *n*_Pop_ = 21). *GRAIN_Y* grain yield, *TGM* thousand grain mass, *EAR_D* single ear density, *KERNELS_E* number of kernels per ear, *FALLING_N* falling number, *PROTEIN_C* crude protein concentration, *AMYLO_V* amylogram viscosity, *AMYLO_T* amylogram temperature, *Hyb* hybrid varieties, *Pop* population varieties. ρ_p_: poolded phenotypic correlation coefficient; ρ_p(Hyb)_, ρ_p(Pop)_: phenotypic correlation coefficient for hybrid and population-type varieties, respectively. *ns* not significant different from zero if *p* > 0.01

Phenotypic and genetic correlation coefficients of winter rye traits are shown in Table 4. Eight phenotypic relations are diagrammatically presented in Fig. 4. Genetic correlation coefficients were calculated according to Eq. (7).

**Table 4:** Phenotypic and genetic correlation for grain and quality traits

		GRAIN_Y	TGM	EAR_D	KERNELS_E	FALLING_N	PROTEIN_C	AMYLO_V	AMYLO_T
Mean	Hyb	91.4	35.9	534.7	50.1	231.4	10.2	841.7	69.6
	Pop	76.3	36.1	503.0	44.0	211.8	10.7	693.1	68.5
GRAIN_Y		1							
		1							
TGM		0.26^ns^	1						
		−0.22^ns^	1						
EAR_D		0.68	−0.25^ns^	1					
		0.55	−0.65	1					
KERNELS_E		0.16^ns^	−0.19^ns^	−0.31	1				
		−0.28^ns^	−0.34	−0.48	1				
FALLING_N		0.39	−0.10^ns^	0.56	−0.15^ns^	1			
		0.34	−0.26^ns^	0.61	−0.37	1			
PROTEIN_C		−0.67	−0.14^ns^	−0.54	0.01^ns^	−0.29^ns^	1		
		−0.82	0.08^ns^	−0.43	0.16^ns^	−0.18^ns^	1		
AMYLO_V		0.33	−0.13^ns^	0.57	−0.23^ns^	0.76	−0.44	1	
		0.39	−0.28^ns^	0.70	−0.49	0.78	−0.41	1	
AMYLO_T		0.51	−0.04^ns^	0.56	−0.05^ns^	0.90	−0.30	0.60	1
		0.34	−0.25^ns^	0.52	−0.31	0.93	−0.13 ^ns^	0.60	1

Mean: average over adjusted means for hybrid (Hyb)- and population (Pop)-type varieties. Upper value: Pooled phenotypic correlation coefficient ρ_p_ after eliminating-type means. Lower value: Genetic correlation coefficient *ρ*_g_. Categorization: 0.25 ≤|*ρ*| < 0.45 weak, 0.45 ≤ |*ρ*| < 0.65 moderate, 0.65 ≤ |*ρ*| < 0.85 strong, 0.85 ≤ |*ρ*| very strong

*GRAIN_Y* grain yield, *TGM* thousand grain mass, *EAR_D* single ear density, *KERNELS_E* number of kernels per ear, *FALLING_N* falling number, *PROTEIN_C* crude protein concentration, *AMYLO_V* amylogram viscosity, *AMYLO_T* amylogram temperature, *Hyb* hybrid varieties, *Pop* population varieties

^ns^Not significantly different from zero if *p* > 0.01

A strong relation was found between grain yield and ear density (*ρ*_p_ = 0.68, *ρ*_g_ = 0.55), a strongly negative with protein concentration (*ρ*_p_ = −0.67, *ρ*_g_ = −0.82), and a positive moderate one with amylogram temperature (Table 4; Fig. 4b, d). No or only a weak association of thousand grain mass with other traits was present (Table 4). Ear density correlates moderately to strongly positively with grain yield and quality traits. Falling number is very strongly correlated with amylogram temperature (*ρ*_p_ = 0.90, *ρ*_g_=0.93, Fig. 4g), strongly with viscosity (*ρ*_p_ = 0.76, *ρ*_g_ = 0.78, Fig. 4f) and moderately with ear density (*ρ*_p_ = 0.56, *ρ*_g_ = 0.61, Electronic Appendix Fig. S1d), but we found only a weak non-significant correlation with protein concentration (*ρ*_p_ = −0.29^ns^, *ρ*_g_ = −0.18^ns^, Fig. 4e). Furthermore, protein concentration is only weakly associated with amylogram viscosity and temperature. Amylogram viscosity and temperature correlate moderately (*ρ*_p_ = 0.60, *ρ*_g_ = 0.60, Fig. 4h). Phenotypic and genetic correlations have the same sign and are of about the same magnitude, except for thousand grain mass and ear density (*ρ*_p_ = −0.25^ns^, *ρ*_g_ = −0.65) (Table 4). When looking at the typewise correlation diagrams in Fig. 4 and Electronic Appendix Fig. S1, it is apparent that for individual traits significant correlation coefficients for hybrid and population varieties have the same sign and are mostly of about the same magnitude.

## Discussion

### Performance progress

#### Grain traits

Results from this study have shown that progress of grain yield for hybrid varieties was much higher than for population varieties. Differences in yield level between hybrid and population varieties increased from 13% (9.2 dt ha^−1^) in 1989 to 18% (15.2 dt ha^−1^) in 2014 relative to yield level of population varieties (Table 3). These differences are in line with figures reported by Hansen et al. (2004) in the range of 10–20%, whereas Miedaner and Huebner (2011) mentioned higher figures (20–25%).

Our results demonstrated further that changes in yield components were due to confounded genetic and non-genetic influences, and showed a different pattern in both groups (Table 3; Fig. 2). A strong increase in ear density for hybrids and a moderate one for population varieties is accompanied by a decline in kernels ear^−1^ in both types, whereas thousand grain mass increased for both types (Table 3). In addition, we derived the single ear mass, i.e., the product of thousand grain mass and number of kernels ear^−1^ to specify the compensating effects of both components. For single ear mass, we found a non-significant gain of 8.6% (0.146 g ear^−1^) for hybrid varieties and a significant increase of 16.4% (0.242 g ear^−1^) for population varieties relative to 1989 (data not shown in Table 3). This demonstrates that progress in thousand grain mass for hybrid varieties was nearly compensated by a reduction of kernels ear^−1^, whereas for population varieties, single ear mass significantly increased and hence compensated more strongly for the apparent decline of kernels ear^−1^. In consequence, progress in grain yield for hybrid varieties is mainly driven by their ability to increase ear density, whereas for population varieties, thousand grain mass was more important than ear density. Dissection of trends in Table 3 shows that for hybrid varieties, genetic effects had a major influence on increased ear density, whereas for population varieties, genetic effects were more important for the observed smaller decline of number of kernels ear^−1^. Increasing sowing rate during the study period can definitely be excluded as reason for increased ear density. In fact, we found that sowing rate in VCU trials was significantly reduced by about 43 kernels m^−2^ during the studied period (Electronic Appendix Table S2). As to our knowledge, on-farm sowing rate decreased parallel to VCU trials, because expert advice to rye growers based on field trials suggested a reduction of seed rate. However, we are not aware of any data-based evidence. Chmielewski and Koehn (2000) and Peltonen-Sainio et al. (2007) supposed that increasing air temperatures caused by global warming, particularly higher temperatures in the autumn and winter time, could have had positive effects on the tillering and thus on the number of ears and thereby on yield level. This is strongly supported by our observations. In Germany, annual average daily air temperatures rose by about 0.9 °C between 1989 and 2014 and sowing dates in VCU trials are about 3 days earlier at the end of the studied period compared to the beginning (Electronic Appendix Table S2). The observed trend towards later autumn sowing dates was likely not mirrored on-farm. During many years, winter cereals were continuously sown earlier to break peak workloads because of increasing farm sizes. Recently, later sowing dates were recommended to farmers because of phythosanitary reasons (virus infection, increasing resistance against grass herbicides). Data-based evidence regarding this questions is not known to us.

#### Quality traits

This study confirms that gain in grain yield and decline in protein concentration had no significant negative influence on indirect quality traits in winter rye which is in agreement with other studies (e.g., Hansen et al. 2004; Muenzing et al. 2014).

Furthermore, we found no significant diverging genetic trends for falling number and amylogram values between hybrid and population varieties in line with Hansen et al. (2004), who stated that the high-yielding hybrid cultivars have starch properties similar to those of population cultivars. In addition, our results indicated a significant genetic progress of hybrid varieties for falling number and amylogram temperature (Table 3; Fig. 2e, h), however, overlaid by non-genetic trends with large standard errors resulting in non-significant overall trends (Table 3; Fig. 1). A noticeable difference occurred with regard to amylogram traits: for hybrids, a gain in amylogram traits was achieved between 1989 and 2014, whereas for population varieties, a loss is indicated, but not significant in both cases (Table 3).

#### On-farm performance

Among cereal crops, winter rye shows the largest gap between VCU trials and national average on-farm yields in Germany (Laidig et al. 2014). The results of our study have shown that in VCU trials, even population varieties are yielding about 55% higher as compared to on-farm yields. This relative gap did not change in magnitude between 1989 and 2014. However, the gap for hybrids widened from 78% in 1989 to 83% in 2014 as compared to on-farm yields (data not shown in Table 3). Even if we take into account that in 2014 still about 19% of national grain harvest was produced from population varieties (Muenzing et al. 2014), our results do suggest a widening yield gap.

Though it is obvious that trial yields are 20–30% higher than on-farm yields, there is still a considerable gap left between VCU trials and on-farm which needs to be explained. As stated by, e.g., Kottmann et al. (2016), rye is mainly grown on less fertile and mostly sandy soils which tend to suffer from water deficiency during the vegetation period. It is likely that average soil conditions and water supply are less favorable under on-farm conditions than in VCU trials when compared with other cereal crops. In addition, on-farm input of fertilizer and fungicides may be suboptimal as compared to VCU trial treatments (Laidig et al. 2014).

For all indirect quality traits, on-farm results showed increasing, though non-significant trends. Bruemmer (2005) compared 2004 with 1960 results of national on-farm averages and found considerable gains of +130 s for falling number, +13 AU for amylogram viscosity, +10 °C for amylogram temperature, and +10 ml for dough yield, but −50 ml for volume yield. He also pointed out that negative effects on crumb elasticity, insufficient bread lightness, and reduced duration of bread fresh keeping may occur if values of indirect quality parameters are very high. This effect is known as “dry baking” and described by Weipert und Zwingelberg (1979). Kucerova (2009) has shown that with increasing falling numbers, the water-unextractable pentosan concentration is increasing too, leading to a suboptimal ratio of unextractable pentosan to total pentosan fraction. An improvement of rye baking quality can be achieved only if a specific ratio exists for water extractable pentosan to total pentosan and of water-unextractable pentosan to starch (Weipert and Zwingelberg 1979).This indicates first that indirect quality traits in this study were not sufficient to predict baking quality fully and, second, that high values of falling numbers do not necessarily mean better baking quality (Weipert 1998b; Oberforster and Werteker 2011). Bruemmer (2005) describes a “quality window” for rye flour with side lengths “falling number” of 120–180 s and “amylogram temperature” of 64–67 °C, within which favorable baking quality could be expected. In many years, national averages for falling number and amylogram temperature were outside the “quality window” (Fig. 1). As only about 15% of the national rye consumption is used for human nutrition, enough rye harvest lots with good baking quality will still be available to cover the annual national demand.

### Genotype and environmental variation

Figure 3 demonstrates that the influence of genotype and environment on yield components for ear density and kernels ear^−1^ is of a similar pattern as that of grain yield: low variance components for genotypes and years, but large variance for locations. The relation for thousand grain mass is reversed, with larger variances for genotypes and years, but lower ones for locations. The relatively high genetic determination of thousand grain mass is in accordance with Chmielewski and Koehn (2000) and with results from a QTL study of Miedaner et al. (2012). This reversed influence of years and location may be explained by the fact that yield components are not equally susceptible to adverse growing conditions. Ear density and kernels ear^−1^ are mostly determined by local growing conditions during autumn and early start of the vegetation, whereas thousand kernel mass is mainly dependent on the more year-related weather condition during grain-filling (Chmielewski and Koehn 2000; Kottmann et al. 2016).

Winter wheat baking quality is mainly determined by protein concentration and quality. Protein-related quality traits in wheat are highly genetically influenced with a genotypic variation of 60–70% relative to total variation (Laidig et al. 2017). In contrast, for winter rye, genetic variation of indirect quality traits is much lower, amounting to between 6% and 19% (Fig. 3). Rye quality is strongly determined by level of alpha-amylase activity measured by falling number (Weipert and Bolling 1979). The dominating effect of year on variability of falling number (41%) and amylogram temperature (50%) indicates a high annual volatility of rye quality. This highly year-dependent nature can be explained mainly by the genetically determined sprouting susceptibility of rye towards wetness, low temperature, and radiation during harvest time, which induces early enzyme activity (Weipert and Bolling 1979). Variation of amylogram viscosity is not as strongly influenced by year (26%) but more by genotypes (19%) as compared to amylogram temperature. Our finding that variation due to genotypes for falling number (8%) and specifically for amylogram viscosity (19%) is larger than for amylogram temperature (6%), is in agreement with results reported by Weipert (1998a) and Kucerova 2009. Weipert (1998a) explained this difference by the fact that viscosity of starch gels reflects the alpha-amylase activity which is indicated by falling number and amylogram results, and that amylogram temperature is related, moreover, with pentosan and starch quality.

### Phenotypic and genetic correlation of grain and quality traits

Ear density showed the strongest phenotypic correlation with grain yield (*ρ*_p_ = 0.68 and *ρ*_g_ 0.55), whereas kernels ear^−1^ and thousand grain mass were not significantly associated with grain yield (Table 4; Fig. 4a–c). We also found only a weak phenotypic correlation of single ear mass with grain yield (*ρ*_p_ = 0.34, *ρ*_g_ = −0.28^ns^, data not shown in Table 4). In accordance with Chmielewski and Koehn (2000), and Kottmann et al. (2016), this again confirms that yield progress in rye is mainly determined by an increase of ear density. Contrary to our result for hybrid varieties, in both studies, a close significant relationship between grain yield and kernels ear^−1^ (*ρ*_p_ = 0.70 and *ρ*_p_ = 0.76, respectively) was found. Our results further showed that phenotypic correlations between yield components were mutually weak and negative; however, a strong significant genetic correlation seems to exist between ear density and thousand grain mass (*ρ*_g_ = −0.65), indicating that both traits are genetically negatively related (Table 3, Electronic Appendix Figs. S1a–c).

Our results confirm the well known and genetically determined negative relation between protein concentration and grain yield (e.g., Simmonds 1995; Hansen et al. 2004; Oberforster and Werteker 2011; Laidig et al. 2017). The negative relation between protein concentration and ear density (*ρ*_p_ = −0.54, *ρ*_g_ = −0.43) can be explained by the strongly positive correlation between grain yield and ear density on the one side and the very strong negative correlation between grain yield and protein concentration on the other side. In contrast to winter wheat, where a strong positive relation between yield and protein exists, our results show that protein concentration and quality traits are only weakly, but negatively related (Table 4; Fig. 4e). This is in accordance with Hansen et al. (2004), who stated that the role of rye protein molecules in relation to bread baking is considered to be low, as no gluten network is formed in rye dough.

It is a great advantage for winter rye breeding that selection towards higher yield and resistance towards sprouting does not affect quality traits negatively, as our results have demonstrated. Wehmann et al. (1991) reported in their study of no serious unfavorable relations between falling number and agronomic traits. Our study showed that there is even a favourable influence on quality traits, as indicated by positive weak-to-moderate correlations between grain yield and falling number, and between amylogram viscosity and temperature (Table 4). In a study of Austrian winter rye VCU trials from 1980 to 2010, including 21 hybrid and 18 population varieties, Oberforster and Werteker (2011) found even stronger positive relations between grain yield and the indirect quality traits falling number and between amylogram viscosity and temperature. Weipert (1998b) explained the positive association of higher yield and increasing trends of quality traits by the fact that besides selection for higher yield, selection was concurrently focused on higher falling numbers. However, higher falling numbers means improved pre-harvest sprouting resistance, and simultaneously increased pentosan concentration. However, as already pointed out, too high falling numbers resulting in an unfavorable pentosan—starch ratio may affect baking quality negatively. Strong-to-very strong correlations were found between falling number and both amylogram values, which is in agreement with the results from other studies on rye quality (e.g., Weipert and Bolling 1979; Rattunde et al. 1994; Hansen et al. 2004). Correlation plots in Fig. 4f–h illustrate the same association patterns for indirect quality traits of hybrid and population varieties, which is in line with Hansen et al. (2004), whose results indicated that the high-yielding hybrid cultivars have starch properties similar to those of population cultivars. The extremely close relationship between falling number and amylogram temperature is not surprising as the expression of both traits is causally related to the level of alpha-amylase activity (Weipert 1983; Rattunde et al. 1994; Bruemmer 2005).

## Conclusions

Enormous progress has been achieved for grain yield in VCU trials of 18.9 dt ha^−1^ for hybrid varieties and 13.0 dt ha^−1^ for population varieties between 1989 and 2014, while the gap between yield level of hybrid and population varieties increased. This study demonstrated that ear density was the determining component for yield progress, whereas for population varieties, thousand grain mass was more important. Yield components were influenced by lower sowing rates, earlier sowing dates, and rising average daily temperatures. In spite of the fact that on-farm share of hybrid varieties continuously increased to 81% in 2014, the yield gap between VCU grain yield and on-farm was 83% for hybrids and 55% for population varieties relative to on-farm level in 2014. Our results revealed that indirect quality traits are subject to large fluctuations from year to year. Thus, in spite of considerable increase in grain yield and decline of protein concentration in VCU trials, indirect quality traits were not significantly positively or negatively affected by increasing grain yields. For on-farm quality traits, we found a positive, but non-significant improvement.

The influence of environment on total variation of grain and quality traits by far exceeds that of genotype and genotype by environment interaction. Only for amylogram, viscosity (19%) and thousand grain mass (10%) were the influence of genotypes of some importance. For quality traits and thousand grain mass, influence of years was the dominating factor for variation. Yet, for grain yield, ear density, and kernels ear^−1^, effects of locations were more important than those of years.

Grain yield was strongly positively correlated with ear density and weakly to moderately positively with falling number and amylogram values, demonstrating that progress in grain yield affected indirect quality traits positively. Falling number correlates very closely with amylogram viscosity (*ρ*_p_ = 0.76) and temperature (*ρ*_p_ = 0.90).

### Author contribution statement

FL conceived the study, carried out the analyses, prepared the figures and tables, and wrote the manuscript. HPP provided advice on statistical analysis, DR in using, and interpreting data. Both read and amended the paper. TD and UM assembled all data sets, prepared, and formatted them for statistical analysis. Both participated in editing the paper. AH was responsible for carrying out laboratory tests for VCU trial and national harvest survey samples and participated in editing the paper.

## Electronic supplementary material

Below is the link to the electronic supplementary material.

Supplementary material 1 (PDF 99 KB)

Supplementary material 2 (PDF 117 KB)
