# Supplementary material for: Breeding progress, variation, and correlation of grain and quality traits in winter rye hybrid and population varieties and national on-farm progress in Germany over 26 years
Source: Theor Appl Genet. 2017 Mar 13;130(5):981–98. doi: 10.1007/s00122-017-2865-9 (PMC5395587; doi:10.1007/s00122-017-2865-9)
Supplement: Supplementary file 1 — Supplementary material 1 (PDF 99 KB) [file 122_2017_2865_MOESM1_ESM.pdf]

Electronic Appendix

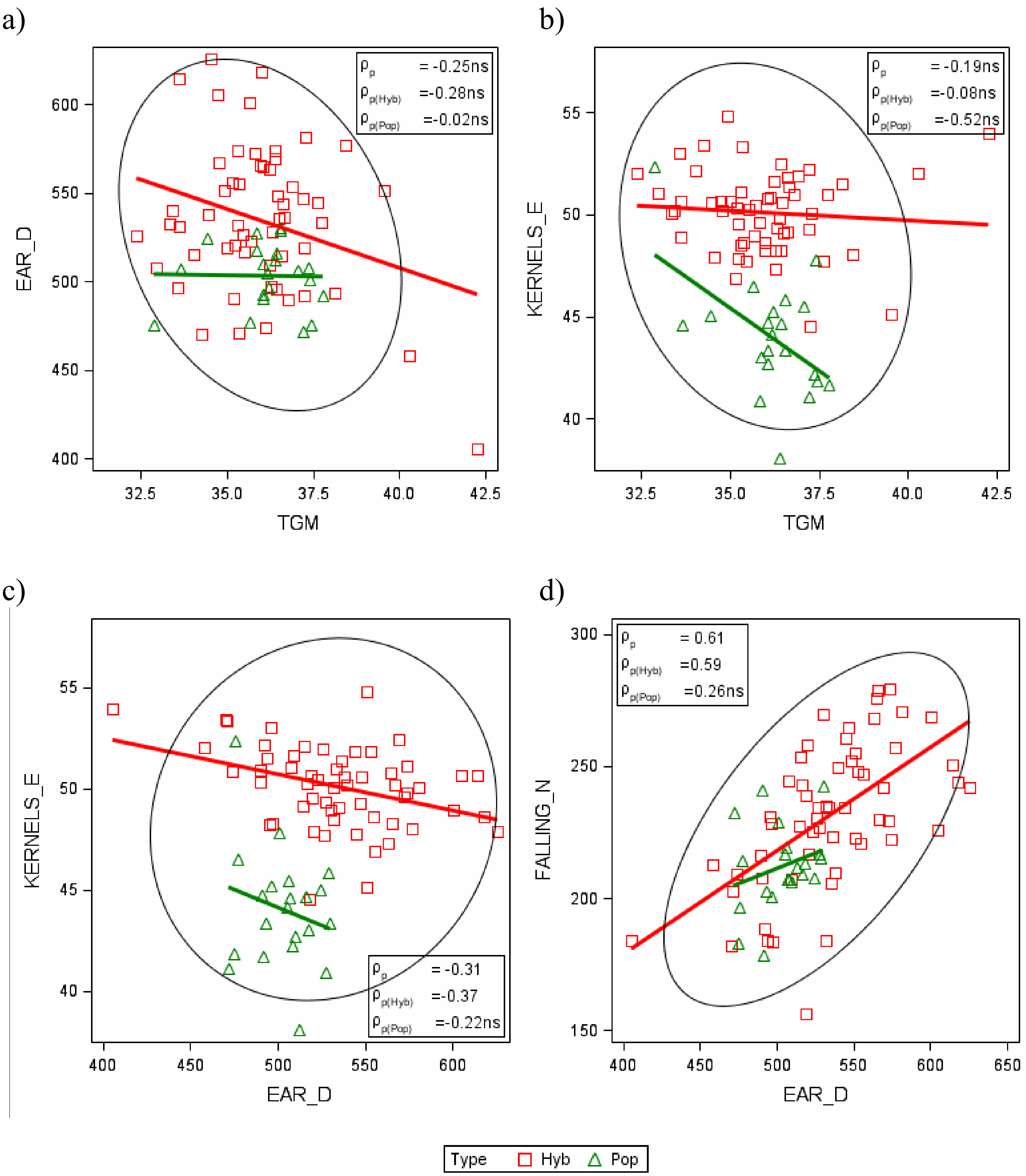

Fig. S1: Correlation of adjusted variety means [effect $G_{i(l)}$ in Eq. (1)] grouped by type of variety, with group regression lines (Number of varieties n_{Hyb} =57 and n_{Pop} = 21).

GRAIN_Y: grain yield; TGM: thousand grain mass; EAR_D: single ear density; KERNELS_E: number of kernels per ear; FALLING_N: falling number;

Hyb: hybrid varieties, Pop: population varieties

ρ_p: poolded phenotypic correlation coefficient; $\rho_{p(Hyb)}$, $\rho_{p(Pop)}$: correlation coefficient for hybrid and population type varieties, respectively

^{ns} not significant different from zero if p>0.01
