# Supplementary material for: Breeding progress, variation, and correlation of grain and quality traits in winter rye hybrid and population varieties and national on-farm progress in Germany over 26 years
Source: Theor Appl Genet. 2017 Mar 13;130(5):981–98. doi: 10.1007/s00122-017-2865-9 (PMC5395587; doi:10.1007/s00122-017-2865-9)
Supplement: Supplementary file 2 — Supplementary material 2 (PDF 117 KB) [file 122_2017_2865_MOESM2_ESM.pdf]

Table S1: Variance components for winter rye grain and quality traits from VCU trials 1989 – 2014. Linear genetic group trends and common linear agronomic trends are eliminated.

Trait	Unit	Variance components						
		Genotype	Year	Location	YxL	GxY	GxL	GxYxL
GRAIN_Y	dt ha^{-1}	3.64	19.19	62.21	100.46	2.26	0.53 ^{ns}	20.41
TGM	g	2.13	5.41	2.49	8.83	0.30	0.11	1.85
EAR_D	m^{-2}	700.57	1,372.57	6,290.11	4,646.78	130.95	84.17 ^{ns}	2,721.24
KERNELS_E	ear^{-1}	2.34	9.73	40.84	29.92	1.38	1.31	26.53
FALLING_N	s	512.49	2645.19	251.30 ^{ns}	2502.16	71.88	45.04	377.20
PROTEIN_C	%	0.074	0.232	0.126 ^{ns}	0.983	0.018	0.002 ^{ns}	0.157
AMYLO_V	AU	27,173	36,660	6,258 ^{ns}	55,290	5,341	1,562	9,180
AMYLO_T	°C	1.54	12.51	0.89 ^{ns}	7.91	0.51	0.09	1.65

^{ns} not significant at 1% level;

AU: amylogram unit

GRAIN_Y: grain yield; TGM: thousand grain mass; EAR_D: single ear density; KERNELS_E: number of kernels per ear; FALLING_N: falling number; PROTEIN_C: crude protein concentration; AMYLO_V: amylogram viscosity; AMYLO_T: amylogram temperature;

Table S2: Change of sowing rate, date of sowing, date of harvest and annual average daily air temperatures during 1988/1989 and 2013/2014 as estimated from regression analyses

Variable	Unit	Data			Regression estimates			Estimate of linear trends		
		Description	Source	Model	1988/1989	2013/2014	Difference	Slope	SE	P value
Sowing rate	Kernels m^{-2}	1094 year-location-combinations (sowing years: 1991-2013)	VCU trials	1	293.4	250.5	–42.9	–1.717	0.170	<.001
Sowing date to 31. Dec.	Days	1094 year-location-combinations (harvesting years: 1992-2014)			88.1	91.1	3.0	0.121	0.053	0.022
01 Jan. to harvesting date	Days	1054 year-location-combinations (harvesting years: 1992-2014)			216.0	215.0	-1.0	–0.039	0.185	0.834
Daily air temperature	°C	45 annual average daily air temperatures in Germany (1970-2014)	DWD 2016 (Deutscher Wettertdienst)	2	8.58	9.46	0.88	0.035	0.010	0.001

DWD (Deutscher Wetterdienst) (2016) German Meteorological Service,

Climate Data Center (ftp://ftp-cdc.dwd.de/pub/CDC/regional_averages_DE/annual/air_temperature_mean/ regional_averages_tm_year.txt). Accessed 12 Oct 2016

Model 1: $y_{jk} = \phi t_k + U_k + L_j + e_{jk}$, where y_{jk} is the observation in jth location and kth year, ϕ is a fixed regression coefficient, t_k is the continuous covariate for the calendar year, U_k is a random deviation of kth year from linear trend, L_j is the random effect of the jth location and ejk is the residual error.

Model 2: $y_k = \mu + \omega t_k + e_k$ where y_k is the annual average daily air temperature in year k, ω is a fixed regression coefficient, t_k represents the calendar year, and e_k is the residual error.

SE: standard error
